# Long-term poor rapport, lack of spontaneity and passive social withdrawal related to acute post-infectious encephalitis: a case report

**DOI:** 10.1186/s40064-016-1994-y

**Published:** 2016-03-18

**Authors:** Atsurou Yamada, Nobuhiro Miyachi, Toshiyasu Miura, Masako Suzuki, Norio Watanabe, Tatsuo Akechi

**Affiliations:** Department of Psychiatry and Cognitive-Behavioral Medicine, Nagoya City University Graduate School of Medical Sciences, Nagoya, Japan; Koseikan Hospital, Ama, Aichi Japan; Department of Neurology, Nagoya City University Graduate School of Medical Sciences, Nagoya, Japan; Department of Health Promotion and Human Behavior, Kyoto University Graduate School of Medicine/School of Public Health, Kyoto, Japan

**Keywords:** Acute disseminated encephalomyelitis, Acute post-infectious encephalitis, Anti-*N*-methyl-d-aspartate receptor encephalitis, Demyelinating disease, Prognosis, Recovery, Psychiatric symptoms

## Abstract

**Introduction:**

Post-infectious encephalitis/encephalopathy is a neurological syndrome that sometimes develops following common viral or bacterial infections. The most common form is acute disseminated encephalomyelitis (ADEM). ADEM is a demyelinating disease of the central nervous system that typically presents as a monophasic disorder associated with multifocal neurologic symptoms and encephalitis. Anti-*N*-methyl-d-aspartate receptor (anti-NMDAR) encephalitis is another type of severe autoimmune disorder, characterized by seizures, movement disorders and psychiatric symptoms. In general, the prognosis and long-term outcomes of both ADEM and anti-NMDAR encephalitis are favorable. Most patients show complete, albeit slow recovery over a period of one to 2 years. There are few reports of patients with these disorders showing long-term residual psychiatric symptoms.

**Case presentation:**

We report the case of a 16-year-old Japanese male who suffered from acute post-infectious encephalitis. The patient followed an atypical recovery course, in that he showed poor rapport, lack of spontaneity and passive social withdrawal for more than 2 years after the initial symptoms. While treatment with small doses of antipsychotic drugs at the hospital had no effect on the symptoms, the patient recovered gradually over a prolonged period of five or so years.

**Conclusions:**

This case report suggests that a type of acute post-infectious encephalitis with demyelinating features, possibly ADEM or anti-NMDAR encephalitis, or an overlap between the two, can cause a prodrome of behavioral changes and long-term residual psychiatric symptoms for many months, although it is eventually associated with a good prognosis.

## Introduction

Post-infectious encephalitis is a neurological syndrome that is known to sometimes develop after common viral or bacterial infections. The most common form is acute disseminated encephalomyelitis (ADEM), which is a demyelinating disease of the central nervous system (CNS) that typically presents as a monophasic disorder associated with multifocal neurologic symptoms and encephalitis. ADEM is considered as an autoimmune disorder. The diagnosis is usually based on the clinical and radiologic features. Lumbar puncture and laboratory studies are useful, however, many children show only non-specific findings of inflammation. Magnetic resonance imaging (MRI) may show widespread, multifocal, asymmetric white- and gray-matter abnormalities, often involving areas of the thalamus or the basal ganglia bilaterally (Menge et al. 2005; Mikaeloff et al. 2007; Gupte et al. 2003; Hynson et al. 2001; Lotze and Chadwick 2011; Dale et al. 2000). Abnormal lesions are typically bilateral, and can be clearly visualized on T2-weighted images and fluid-attenuated inversion recovery (FLAIR) images. The number and sizes of the lesions vary among patients. The differential diagnosis includes such conditions as multiple sclerosis, optic neuritis, transverse myelitis, neuromyelitis optica, infectious meningoencephalitis, anti-phospholipid antibody syndrome, isolated CNS angiitis, vasculitis secondary to rheumatic autoimmune disease, CNS neoplasms, neurosarcoidosis, mitochondrial encephalopathies, and adrenoleukodystrophy (Lotze and Chadwick 2011; Menge et al. 2005; Tenembaum et al. 2007). The prognosis and long-term outcome of ADEM are favorable.

On the other hands, anti-*N*-methyl-d-aspartate receptor (anti-NMDAR) encephalitis is another type of severe autoimmune disorder developing following common viral/bacterial infections, that is characterized by seizures, movement disorders and psychiatric symptoms. Brain MRI in cases of anti-NMDAR encephalitis is frequently normal or may reveal only subtle lesions, not usually restricted to the white matter. Patients with anti-NMDAR encephalitis may be diagnosed as having different conditions at different stages of the disease. New-onset psychosis or viral encephalitis is an early presumptive diagnosis in some patients. Anti-NMDAR encephalitis patients often show spontaneous neurological improvement, although the recovery can be incomplete or delayed. Some patients show social-behavioral abnormalities and impaired executive functions for many months (Dalmau et al. 2011).

Herein, we describe the atypical case of a 16-year-old male patient who was diagnosed as having acute post-infectious encephalitis, and presented with psychiatric symptoms such as poor rapport, poor motivation and flattened affect that persisted for a long period of time after recovery from the initial symptoms.

## Case presentation

The patient was a 16-year-old Japanese male, with a normal birth history and normal developmental milestones. He was a high-achieving pupil, with no family history of psychiatric disorders. He had received his last diphtheria/pertussis/tetanus and measles vaccinations at the age of one and a half years, with no history of any significant health problems thereafter.

**Fig. 1:**
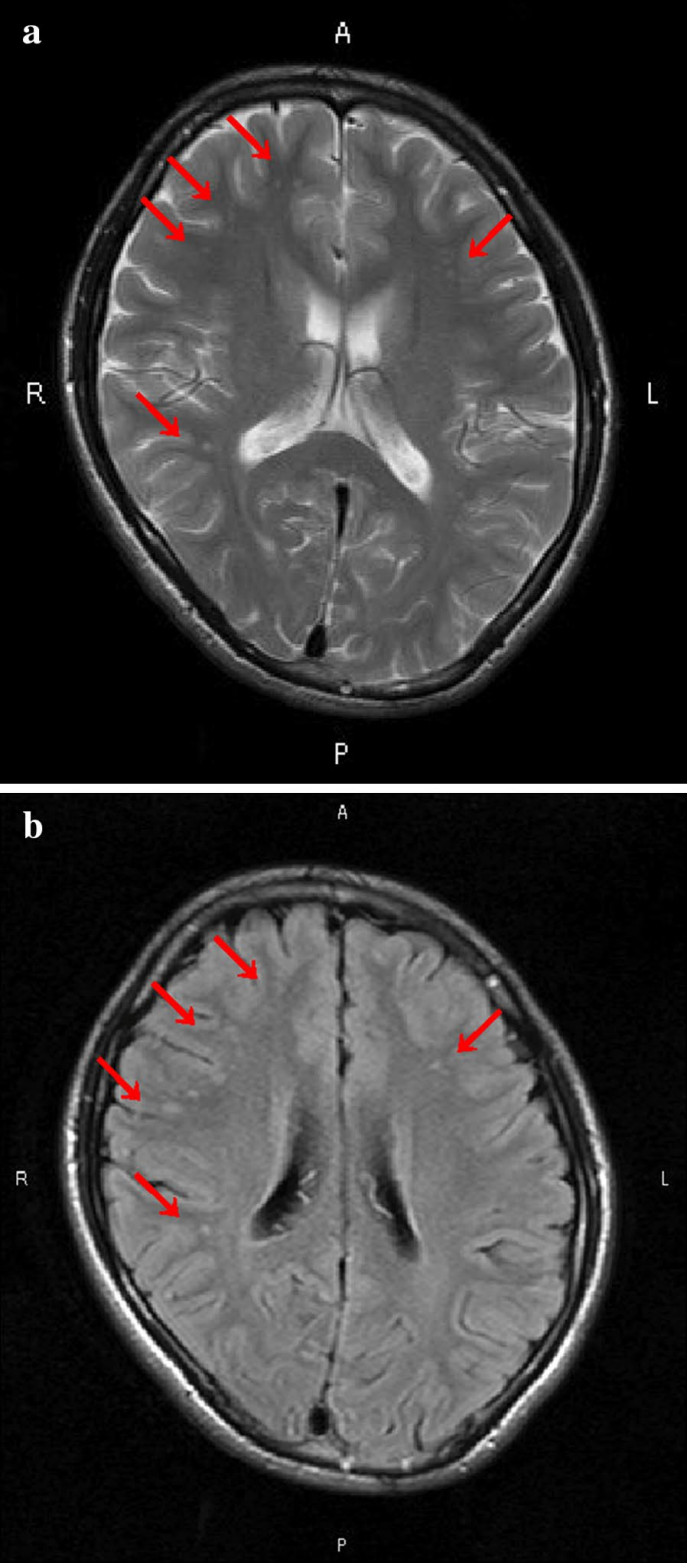
Magnetic resonance imaging (MRI) of the brain: **a** T2-weighted image. **b** fluid-attenuated inversion recovery (FLAIR) image

When he was 15 years old, the patient began to develop a defiant attitude towards his father. He often stayed up late using the Internet and his school grades began to deteriorate. There were no prodromal symptoms at that time. At the start of the present illness, the patient developed easy fatigability with throat and foot pain. It was thought that the symptoms were related to a cold, and he attended school after taking medicines for common cold. Ten days later, however, he stopped eating and responding to others. Thereafter, after 2 days, he developed fever and quivered convulsively, and his parents took him to a neighborhood hospital. MRI of the brain was performed, and the T2-weighted and FLAIR images revealed high-intensity lesions in the subcortical white matter of the frontal lobes bilaterally, and in the left temporal lobe (Fig. 1a, b). These lesions on the T2 and FLAIR images could not be visualized on the T1 sequences. Cerebrospinal fluid (CSF) examination revealed the following: cell count 66/mm^3^ (monocytes 43/mm^3^), protein 41.5 mg/dl, glucose 81 mg/dl. Based on a suspicion of viral encephalitis, the patient was started on an antiviral drug and steroid pulse therapy. The second CSF examination revealed a cell count of 8/mm^3^ (monocytes 3/mm^3^), protein level of 25.9 mg/dl, negative results for oligoclonal bands and myelin basic protein, and normal levels of IgG and IgM. The patient’s level of consciousness improved the following day, and with the resolution of all the other symptoms a week later, he was discharged. The following month, the patient gradually stopped going to school and began to stay at home. He also refused to go to the hospital. A month later, his family managed to bring him to the Neurology Department of our hospital. After the initial examination, the neurologist recommended hospitalization for more detailed examination, however, the patient refused. The doctor then referred him to our psychiatry department.

**Fig. 2:**
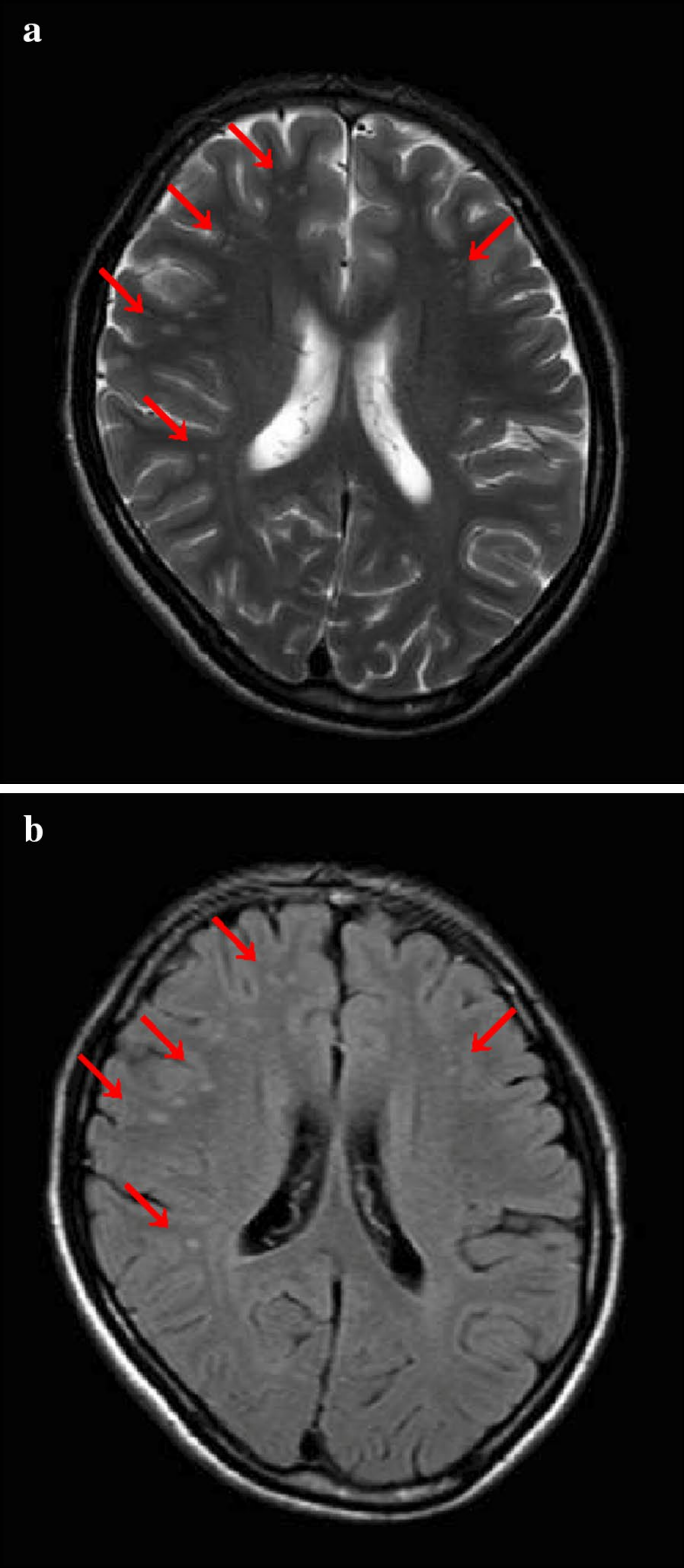
Magnetic resonance imaging (MRI) of the brain after hospitalization: **a** T2-weighted image. **b** Fluid-attenuated inversion recovery (FLAIR) image

When he attended his first appointment, he was on a wheelchair. He did not answer any questions, keeping his head low and his eyes shut. Assessment on the Glasgow Coma Scale revealed a score of 4 for eye opening, 4 for verbal response and 6 for motor response, and it was not clear why he was refusing to talk or engage in conversations. With his parents’ consent, we decided to hospitalize him for closer examination. He did not resist, but he continued to refuse to communicate with the hospital staff even after hospitalization. He had no paralysis, no finger tremors, or any neck stiffness. The muscle tone and reflexes could not be clearly assessed, because the patient’s refusal to cooperate. He could eat and take care of his personal activities of daily living, although he was unwilling to take a bath daily. Rheumatologic disease, such as CNS lupus or vasculitis, was excluded, because the serological tests for antinuclear antibody, Rh factor, antimitochondrial antibody, proteinase-3 anti-neutrophil cytoplasmic antibody (PR3-ANCA) and myeloperoxidase anti-neutrophil cytoplasmic antibody (MPO-ANCA) were negative. Other antibodies associated with some other forms of autoimmune encephalitis, e.g. anti-NMDAR and anti-voltage gated potassium channel antibodies, were not tested for. The same high-intensity lesions in the subcortical white matter as those seen a month earlier were still observed on the T2-weighted and FLAIR images on brain MRI (Fig. 2a, b).

**Fig. 3:**
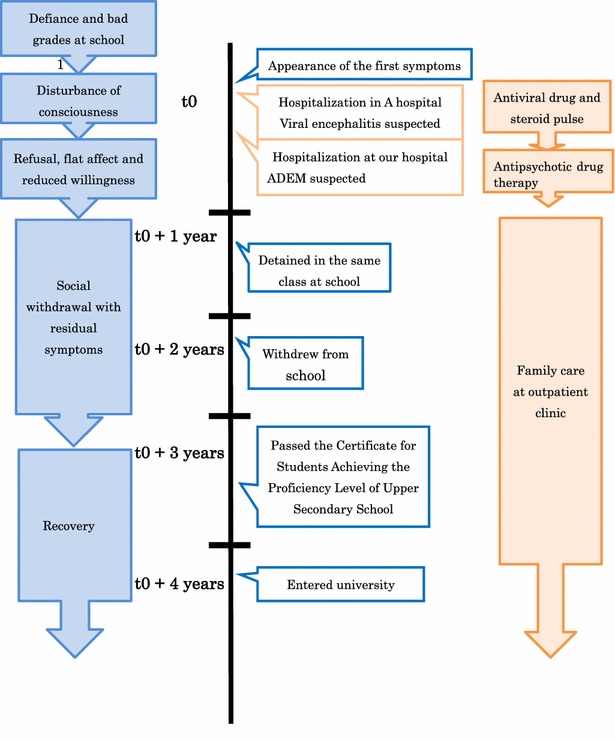
Timeline of interventions and outcomes. ADEM: acute disseminated encephalomyelitis

After admission, the patient isolated himself in his room and refused to talk, not only to the medical staff, but also to his own family members. We could not assess whether he suffered from disorientation and speech impediment, although he took his meals regularly and slept well. The electroencephalogram showed neither slow nor spike waves. We considered the possibility of disorganized schizophrenia, because of his disorganized behavior and speech, flat affect, and disturbance in behavior, communication and thought. We initiated treatment with risperidone, at a starting dose of 0.5 mg daily, which was gradually increased to 4 mg over a period of 4 weeks. Because there was no effect, however, we added aripiprazole at the dose of 6 mg daily for 2 weeks, however, this also had no effect on the patient’s symptoms. One month after admission, the patient began to speak gradually, and demanded to leave the hospital. When we explained the need for further assessment of his health status, he agreed, and an intelligence assessment, lumbar puncture, and single photon emission computed tomography (SPECT) were performed. SPECT showed slightly enhancing lesions in the anterior lobes, which were non-specific findings. CSF examination revealed the following findings: cell count 0/mm^3^, protein 18 mg/dl, glucose 64 mg/dl, IgG, IgA and IgM levels within normal limits. Examination by the Wechsler Intelligence Scale for Children—Third Edition (WISC-III) showed an above-average Intelligence Quotient (IQ). The patient was discharged from the hospital, but still tended to stay at home and absent himself from school. Even when he tried to attend school, he could not persist with it. At home, he took care of his personal activities of daily living, but could not sleep regularly and often became depressed. We increased the dose of aripiprazole to 12 mg daily and decreased the dose of risperidone to 3 mg for 2 weeks after discharge. We suggested continuing the antipsychotic drug treatment to him and his mother, but both disagreed and the treatment was stopped. Nine months later, he was unable to proceed to the next year of school and 2 years later, he quit high school. He was not sociable, and sometimes showed depressed mood. A repeat brain MRI at that point revealed no changes from the findings at the earlier imagings. However, thereafter, the patient gradually recovered, and about 2 years later, he passed the Certificate for Students Achieving the Proficiency Level of Upper Secondary School Graduates. During the entire course, the patient showed no evidence of auditory hallucinations, delusions or other symptoms suggestive of schizophrenia. Overall, his presentation remained stable during follow-up. The timeline is shown in Fig. 3.

## Discussion

The diagnosis in this patient was first suspected as ADEM, because (1) the clinical symptoms, such as disturbance of consciousness and convulsions, developing after the infectious symptoms were suggestive of acute encephalitis, (2) there were disseminated demyelinating lesions on head MRI, and (3) there were no signs suggestive of viral encephalitis, bacterial meningitis or other immunologic diseases such as CNS lupus or vasculitis.

The patient followed an atypical recovery course, in that he showed poor rapport, lack of spontaneity and passive social withdrawal for more than 2 years after the initial symptoms. He did not have any paralyses or other neurological symptoms, and his IQ was above average. The long-term clinical outcome in patients with ADEM is usually favorable. Most children show complete, although slow, recovery over a period of 1–6 months. According to previous reports, about 57–89 % of cases show full recovery with few residual deficits (Hynson et al. 2001; Menge et al. 2005; Nasr et al. 2000; Lotze and Chadwick 2011; Tenembaum et al. 2007). However, there are also reports of cases not showing full recovery and manifesting residual symptoms. Such patients could show severe symptoms, such as hemiparesis, partial seizures, reduction of visual acuity, paraparesis, urinary retention, neuropathic bladder, dysphasia, behavioral problems or mental handicap (Murthy et al. 2002; Dale et al. 2000; Tenembaum et al. 2002). Moreover, subtle neurocognitive deficits in attention, executive function, behavioral changes and psychiatric disturbances and speech disturbance have been demonstrated, even after 3 years, in some children who were thought to have recovered fully (Suppiej et al. 2014; Tenembaum et al. 2007; Hynson et al. 2001; Hahn et al. 2003). Onset of illness after 6 years of age has also been shown to be associated with decreased Intelligence Quotient scores in verbal processing (Jacobs et al. 2004). In our patient reported here, the findings of high signal intensity lesions on the T2-weighted and FLAIR images on head MRI remained unchanged even after the patient recovered from the clinical symptoms. His recovery course after acute post-infectious encephalitis was consistent with that reported for cases of ADEM.

This patient had some atypical features of ADEM: (1) the high signal intensity lesions on MRI were smaller than the typical lesions of ADEM; (2) the imaging findings did not improve with convalescence; (3) the patient did not have a polysymptomatic presentation. Thus, the possibility of another type of acute post-infectious encephalitis cannot be excluded. Autoimmune encephalitis cannot be excluded because tests for antibodies such as anti-NMDAR and anti-voltage gated potassium channel antibodies were not performed. This patient showed behavioral changes, psychiatric disturbances and might have even had speech disturbance, which suggest the possibility of anti-NMDAR encephalitis. It has now been reported that patients with anti-NMDAR encephalitis may develop demyelinating features as concurrent or independent episodes and in some cases, anti-NMDAR encephalitis has been diagnosed after ADEM. Anti-NMDAR encephalitis is frequently associated with a normal brain MRI, despite its clinical severity, which remains a clinico-radiologic paradox. Patients with this condition often show psychiatric symptoms at the onset (Dalmau et al. 2011; Sartori et al. 2015). If left untreated, patients typically recover spontaneously after many years. Patients suffering from demyelinating disorders with prominent psychiatric manifestations may have NMDAR encephalitis (Titulaer et al. 2014; Hacohen et al. 2014). There is a possibility that our patient suffered from anti-NMDAR encephalitis which caused the prodrome of rebellious behavior and residual psychiatric illness. Moreover, this patient might have suffered from catatonia, which is a feature of anti-NMDAR encephalitis, during his last hospitalization. In these cases, antibody studies are necessary to confirm the diagnosis of anti-NMDAR encephalitis, but it is difficult to distinguish between ADEM and anti-NMDAR encephalitis, and the two conditions may overlap. The frequencies of these disorders are not yet known (Titulaer et al. 2014). Finally, we hypothesized that the entire course of the disease in our patient, including the prodrome of rebellious behavior, the acute encephalitis and the residual psychiatric syndrome, was consistent with the diagnosis of ADEM or anti-NMDAR encephalitis, or an overlap between the two conditions.

The strength of this report lies in that we could examine the patient closely, could assess his psychiatric symptoms during hospitalization, and also follow the patient up for a prolonged period of time after he was discharged from the hospital. However, there were some limitations. First, the diagnosis of the acute post-infectious encephalitis could not be definitively established, because testing for other autoimmune encephalitides, especially NMDAR encephalitis, were not undertaken. Second, the neurological data and clinical information were not sufficient and remained unclear, because the patient was treated at another hospital during the acute phases. Furthermore, case studies of various acute post-infectious encephalitides are warranted.

## Conclusions

Our patient reported here suffered from acute post-infectious encephalitis and showed an atypical recovery course, manifesting psychiatric symptoms, including poor rapport, lack of spontaneity and passive social withdrawal for a period of over 2 years after the initial symptoms. There were no significant neurological manifestations such as paralysis, epilepsy or visual impairment, and the patient eventually showed a good outcome. We hypothesized that the entire clinical course was consistent with the diagnosis of ADEM or anti-NMDAR encephalitis, or an overlap between the two conditions.

## Abbreviations

ADEM: acute disseminated encephalomyelitis
anti-NMDAR: anti-*N*-methyl-d-aspartate receptor
CNS: central nervous system,
CSF: cerebrospinal fluid
FLAIR: fluid-attenuated inversion recovery
SPECT: single photon emission computed tomography
MRI: magnetic resonance imaging
PR3-ANCA: proteinase-3 anti-neutrophil cytoplasmic antibody
MPO-ANCA: myeloperoxidase anti-neutrophil cytoplasmic antibody
WISC-III: Wechsler Intelligence Scale for Children—Third Edition
IQ: Intelligence Quotient
